# Comparative Study on High Strain Rate Fracture Modelling Using the Application of Explosively Driven Cylinder Rings

**DOI:** 10.3390/ma14154235

**Published:** 2021-07-29

**Authors:** Marvin Becker, Tom De Vuyst, Marina Seidl, Miriam Schulte

**Affiliations:** 1 Institute of Saint-Louis, 5, rue du Général Cassagnou, 68301 Saint Louis, France; marina.seidl@isl.eu; 2 Department of Engineering and Technology, University of Hertfordshire, Hatfield AL10 9AB, Hertfordshire, UK; t.de-vuyst@herts.co.uk; 3 Institute for Parallel and Distributed Systems, University of Stuttgart, Universitätsstr. 38, 70569 Stuttgart, Germany; miriam.schulte@ipvs.uni-stuttgart.de

**Keywords:** constitutive modelling, damage modelling, smoothed-particle hydrodynamics, highly dynamic expansion, fragmentation

## Abstract

The effect of different constitutive modelling choices is crucial under a high strain rate as encountered in ballistic applications. Natural fragmentation of explosively driven cylinder rings is chosen as a simplified example to describe the ability of numerical simulations to describe fractures. The main research interests are the importance of (i) material imperfections, (ii) the accuracy of fracture models vs. damage models, (iii) the plasticity algorithm (stress update), (iv) the introduction of a triaxiality cutoff criterion to the damage models, and (v) different constitutive models (plasticity and damage). Due to the complexity of the propagation and coalescense of multiple cracks in classical methods, smoothed-particle hydrodynamics (SPH) is used as a tailor-made method to discretise the model. An elasto-plasticity model, a damage model and an equation of state describe the material behaviour. The required material parameters are determined based on stress–strain curves from quasi-static and dynamic tests. The Johnson–Cook model, with and without a modification of the strain rate term, and the Rusinek–Klepaczko model are used to describe plasticity. These plasticity models are combined either with the Johnson–Cook, the Lemaitre, or the Dolinski–Rittel damage model and the Mie–Grüneisen equation of state. The numerical results show that (i) a random distribution of initial damage increases irregularity of cracks, and gives more realistic fragment shapes, (ii) a coupling of plasticity model and fracture criterion has only a small effect on the fracture behaviour, (iii) using an iterative plasticity solver has a positive effect on the fracture behaviour, although this effect is marginal, (iv) adding a triaxiality cutoff criterion to the damage models improves the predicted fragment masses in the numerical simulations significantly, and (v) good accordance between experiments and numerical simulations are found for the Dolinski–Rittel and Lemaitre damage model with both plasticity models.

## 1. Introduction

Fragmentation of cylinder rings is a demonstrative application where a high strain rate fragmentation of ductile materials occurs. Fast compression or expansion can provoke the break up of the ring. In experiments, this can be induced by electromagnetic forces that are generated by discharging a capacitor; or by pressure forces due to the detonating of an explosive. Grady and Benson [1], and others [2,3,4] compare electromagnetically driven expanding ring experiments and Olovsson et al. [5] investigate electromagnetically driven compression. Our study investigates ring expansion due to the detonation of an explosive that generates characteristic fragments. The idea of looking at the fragmentation of cylinder rings goes back to the pioneering work of Mott [6]. Since then, many authors investigated fragmentation of cylinder rings under radial expansion analytically and numerically to study fracture of ductile material at high strain rates [3,7,8,9,10,11,12,13,14,15,16,17,18,19,20].

The experiments for our study have been conducted at ISL and published by De Vuyst et al. [21]. Furthermore, ref. [21] presents numerical results for this application. The main drawbacks of the model are the description of circumferential cracks and the fragment mass distribution for the smallest ring size. Becker et al. [22] studied different numerical formulations and material parameters in *LS-DYNA^®^* for this application for one single ring size to evaluate the SPH capabilities of *LS-DYNA^®^*.

This paper focuses on all ring sizes and introduces new modelling techniques. Section 2 discusses the relevant methodology, including SPH, plasticity, and fracture models. Section 3 describes the determination of the material parameters. Section 4 illustrates the application and the results obtained with the improved numerical model. This model gives a better description of qualitative aspects compared to the original model [21]. e.g., the fragment mass distribution matches better for all ring sizes and the second fragment layer for larger ring sizes, observed in the experiments, is predicted by our model.

## 2. Numerical Methods and Identification of Material Parameters

### 2.1. Smoothed-Particle Hydrodynamics (SPH)

**Figure 1:**
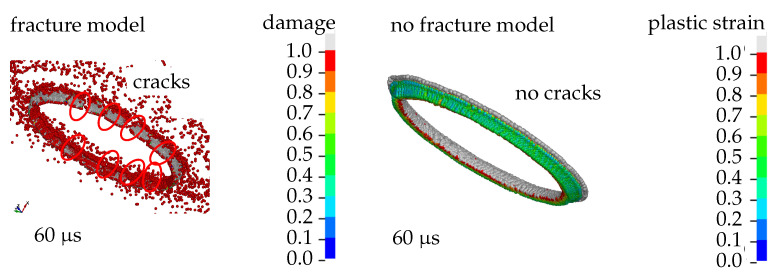
Cracks are visible only when the fracture model is enabled. Otherwise, the ring stays intact until the particles loose contact.

SPH is a meshless method to approximate the solution of partial differential equations. The family of meshless methods is, besides many other applications, of particular interest for the prediction of fracture and fragmentation at high strain rates in metals. Meshless methods can deal with large deformations as well as propagation, bifurcation, and joining of cracks. In contrast, mesh-based methods, such as the Finite Element Method (FEM), need additional modeling techniques, such as node-splitting or erosion criteria, to represent cracks that occur during natural fragmentation. Furthermore, large deformations decrease the accuracy of standard element formulations and increase the run time. Initially, the SPH method was developed for astrophysics problems by Lucy [23] and Gingold [24]. Later, Libersky and Petschek et al. [25,26] extended SPH to deal with materials with strength. The discretized conservation equations used in this paper are

(1) $$\begin{array}{cc} \frac{d\rho_{a}}{dt} & =\rho_{a}\sum_{b}^{}\frac{m_{b}}{\rho_{b}}\left(v_{a}-v_{b}\right)\nabla_{a}W_{ab},\\ \frac{dv_{a}}{dt} & =-\sum_{b}^{}m_{b}\left(\frac{\sigma_{a}}{\rho_{a}^{2}}+\frac{\sigma_{b}}{\rho_{b}^{2}}\right)\nabla_{a}W_{ab}+f_{b}W_{ab},\;\mathrm{and}\\ \frac{dE_{a}}{dt} & =-\frac{\sigma_{a}}{\rho_{a}^{2}}\sum_{j}m_{j}\left(v_{a}-v_{b}\right)\nabla_{a}W_{ab}, \end{array}$$

where $\rho_{a}$ denotes the density, $v_{a}$ the velocity, $E_{a}$ the internal energy, $m_{a}$ the mass, and $\sigma_{a}$ the stress of a particle *a*. $W_{ab}$ is the so-called kernel function *W* evaluated at the distance $\left|\right|x_{a}-x_{b}\left|\right|$ between two particles *a* and *b*, $f_{b}$ are the body forces and $\nabla_{a}$ is the gradient with respect to $x_{a}$. The standard SPH formulation presented in Section 2.1 suffers from a numerical instability [27]. Therefore, total Lagrangian descriptions that overcome the problem have been developed in the recent years [28,29]. However, these kinds of formulations are only applicable to moderate strains [30]. Thus, a total Lagrangian description poses numerical difficulties for our application. Eulerian methods, on the other hand, are able to deal with large deformations and fracture [31,32]. We assured by using a Monaghan bond viscosity [33] that tensile instability is not influencing the solution in our application (compare Figure 1).

Our SPH solver integrates these equations with a central difference scheme in time [4,34].

In the following subsections, we present the constitutive models to describe σ in (1).

### 2.2. Modeling Metal Plasticity

We use the isotropic metal plasticity by the von-Mises yield criterion, which is combined with a hardening rule that specifies the yield strength $\sigma_{y}$ for a particular loading condition at each point. Natural fragmentation includes high strain rates, which are only considered by particular hardening rules. A previous study indicates that the Cowper–Symonds modification in the Johnson–Cook hardening rule improves the qualitative description of natural fragmentation [21]. Since this modification adds another empirical parameter to the model, we aim for a model which is based on physical rules and requires less parameters to fit, such as the Rusinek–Klepaczko model. Furthermore, the Rusinek–Klepaczko model is supposed to be accurate over a wider range of strain, strain-rates, and temperatures. Consequently, we compare the following three hardening rules: the Johnson–Cook model (with and without a modification of the strain rate term), and the Rusinek–Klepaczko model [12]. A Mie–Grüneisen equation of state is combined with the elasto-plasticity model to capture the large pressure waves due to the explosive loading. Among the plasticity models, the Rusinek–Klepaczko model has not been combined with an equation of state so far [12].

#### 2.2.1. Johnson–Cook (JC) Model

The JC model [35] expresses the hardening in terms of effective plastic strain ${\bar{\varepsilon }}_{p}$, plastic strain rate ${\dot{\bar{\varepsilon }}}_{p}$, and current temperature *T*

(2) $$\sigma_{y}\left({\bar{\varepsilon }}_{p},{\dot{\bar{\varepsilon }}}_{p},T\right)=\left(A+B{{\bar{\varepsilon }}_{p}}^{n}\right)\left(1+C\ln({\dot{\varepsilon }}^{*})\right)\left(1-{\left(T^{*}\right)}^{m}\right)$$

where *A* is the yield stress at ambient conditions, B,C,n, and *m* describe other input constants,

(3) $${\dot{\varepsilon }}^{*}=\frac{{\dot{\bar{\varepsilon }}}_{p}}{{\dot{\varepsilon }}_{0}}$$

is the effective plastic strain rate normalized with the testing strain rate ${\dot{\varepsilon }}_{0}$, and

(4) $$T^{*}=\frac{T-T_{r}}{T_{m}-T_{r}}$$

is a homogenized temperature, defined by the melting temperature $T_{m}$ and room temperature $T_{r}$. To account for an adiabatic temperature increase, the temperature is updated by the Taylor–Quinney equation

(5) $$T=T_{0}+\frac{\beta }{\rho C_{p}}\int_{0}^{{\bar{\varepsilon }}_{p}}\sigma \left({\bar{\varepsilon }}_{p},{\dot{\bar{\varepsilon }}}_{p},T\right)d{\bar{\varepsilon }}_{p},$$

where $T_{0}$ is the initial temperature, ρ is the density, $C_{p}$ is the specific heat, and β is the Taylor–Quinney coefficient which may vary with plastic deformation [36], but is assumed to be a constant β=0.9 in our work (e.g., Nahson et al. [37]).

#### 2.2.2. Cowper-Symonds (CS) Modification of the JC Model

The CS model accounts for a nonlinear change of the strain rate hardening. Here, the modification is applied to the JC model resulting in a modified JC model. Instead of the term $\left(1+C\ln({\dot{\varepsilon }}^{*})\right)$ in (2), the scaling factor for the CS model reads

(6) $$1+{\left(\frac{{\dot{\varepsilon }}^{*}}{\bar{C}}\right)}^{\frac{1}{\bar{p}}}$$

where $\bar{C}$ and $\bar{p}$ are material specific constants. The modified equation is

(7) $$\sigma_{y}\left({\bar{\epsilon }}_{p},{\dot{\bar{\varepsilon }}}_{p},T\right)=\left(A+B{{\bar{\epsilon }}_{p}}^{n}\right)\left(1+{\left(\frac{{\dot{\varepsilon }}^{*}}{\bar{C}}\right)}^{\frac{1}{\bar{p}}}\right)\left(1-{\left(T^{*}\right)}^{m}\right).$$

According to the experimental data published by Meyers [38] and Lee et al. [39], the empirical formula describes the material behaviour of metals better at very high strain rates (>1000 s${}^{-1}$).

#### 2.2.3. Rusinek–Klepaczko (RK) Model

The RK model consists of two physics-based components: The first one is the internal stress $\sigma_{\mu }$ that describes the creation of new immobile dislocations which lead to strain hardening, and the second one is the effective stress $\sigma^{*}$ that models the thermal activation process. The yield stress is defined as follows:

(8) $$\sigma_{y}\left({\bar{\varepsilon }}_{p},{\dot{\bar{\varepsilon }}}_{p},T\right)=\frac{E(T)}{E_{0}}\left(\sigma_{\mu }\left({\bar{\varepsilon }}_{p},{\dot{\bar{\varepsilon }}}_{p},T\right)+\sigma^{*}\left({\bar{\varepsilon }}_{p},{\dot{\bar{\varepsilon }}}_{p},T\right)\right),$$

where *E* is the evolution of Young’s modulus as a function of temperature softening, and $E_{0}$ is the Young’s modulus at initial conditions. The expression for the evolution of *E* is based on physical considerations [40] and reads

(9) $$\frac{E(T)}{E_{0}}=1-\frac{T}{T_{m}}\exp\left(\frac{T_{ch}}{T_{m}}\left(1-\frac{T_{m}}{T}\right)\right),$$

where $T_{m}$ is the melting temperature and $T_{ch}$ is a characteristic temperature. Note that the temperature normalization is different from the definition in the JC model (compare (4)). For the internal and effective stress components, the following expressions are used:

(10) $$\sigma_{\mu }=B\left({\dot{\bar{\varepsilon }}}_{p},T\right){\left(\varepsilon_{0}+{\bar{\varepsilon }}_{p}\right)}^{n({\dot{\bar{\varepsilon }}}_{p},T)},$$

with internal stress *B* and strain-exponent *n* described in the following, and

(11) $$\sigma^{*}=\sigma_{0}^{*}{\left[1-{\tilde{D}}_{1}\frac{T}{T_{m}}\log\left(\frac{{\dot{\varepsilon }}_{\max}}{{\dot{\bar{\varepsilon }}}_{p}}\right)\right]}^{m},\;\sigma^{*}\geq 0,$$

where $\sigma_{0}^{*}$ is the initial effective stress, ${\tilde{D}}_{1}$ is a material specific constant, ${\dot{\varepsilon }}_{\max}$ defines a case specific upper bound of the strain rate, and $\sigma_{0}$ is the effective stress at T=0K. The constant ${\tilde{D}}_{1}$ is a dependent parameter and can be identified as follows: the stress component $\sigma^{*}$ vanishes when the critical temperature is reached

(12) $${\tilde{D}}_{1}\frac{T_{c}}{T_{m}}\log\left(\frac{{\dot{\varepsilon }}_{\max}}{{\dot{\varepsilon }}_{\min}}\right)=1,$$

where $T_{c}$ is the critical temperature (in practice, the room temperature). The second stress term $\sigma^{*}$ is also physics-based as it is similar to the equation of Arrhénius [41], which describes the kinetics of thermally activated processes. The internal stress components *B* and *n* are described by the following two equations:

(13) $$B\left({\dot{\bar{\varepsilon }}}_{p},T\right)=B_{0}{\left[\frac{T}{T_{m}}\log\left(\frac{{\dot{\varepsilon }}_{\max}}{{\dot{\bar{\varepsilon }}}_{p}}\right)\right]}^{-\nu },\;\mathrm{and}$$

(14) $$n\left({\dot{\bar{\varepsilon }}}_{p},T\right)=n_{0}\left(1-{\tilde{D}}_{2}\frac{T}{T_{m}}\log\left(\frac{{\dot{\bar{\varepsilon }}}_{p}}{{\dot{\varepsilon }}_{\min}}\right)\right)\;n\left({\dot{\bar{\varepsilon }}}_{p},T\right)\geq 0,$$

where $n_{0}$ is the strain hardening exponent at T=0 K, ${\tilde{D}}_{2}$ is a material constant, ${\dot{\varepsilon }}_{\min}$ is the minimum strain rate assumed in the model, $B_{0}$ is the so-called plasticity modulus at T=0 K, and the constant ν characterizes the temperature sensitivity of flow stress. The model parameters are only given for T=0 K but estimated from tests at ambient conditions. If a negative value is computed for *n*, it is set to 0. To account for an adiabatic temperature increase, we update the temperature similar to the JC model with the Taylor–Quinney equation (Equation (5)).

### 2.3. Modeling Damage Accumulation of Metals

A damage model consists of two parts: a fracture criterion, which accumulates an internal damage variable *D* until a threshold value $D_{c}$, and a relation between *D* and the yield stress. When the fracture criterion

(15) $$D=D_{c}$$

is fulfilled, the material fails and the off-diagonals of the stress tensor are set to zero. By this, the material is not able to respond to external loading. Consequently, the failed particles behave like a fluid and can still exchange momentum, but are not connected to other particles anymore. The following equation describes the accumulation of damage *D* for the effective plastic strain ${\bar{\varepsilon }}_{p}$

(16) $$D=\int_{0}^{{\bar{\varepsilon }}_{p}}\dot{D}\left(\sigma_{i},\dot{\varepsilon },T,\ldots \right)d{\bar{\varepsilon }}_{p}$$

where $\sigma_{i}$ are stress components or stress invariants of σ, $\dot{\varepsilon }$ is the strain rate and *T* the temperature. The stress state includes, in particular, triaxiality $\sigma^{*}$, which is the ratio of the hydrostatic pressure or mean stress $\sigma_{m}$ to the equivalent von Mises stress $\sigma_{eq}$

(17) $$\sigma^{*}=\frac{\sigma_{m}}{\sigma_{eq}}.$$

Depending on the choice of $\dot{D}$, we obtain different types of ductile fracture criteria; e.g., the Johnson–Cook (compare Section 2.3.1), the Lemaitre (compare Section 2.3.2), and the Dolinski–Rittel model (compare Section 2.3.3).

#### 2.3.1. Johnson–Cook Fracture Criterion (JCf)

The JC fracture model is a well-known model for high velocity impact [42]. It calculates

(18) $$\dot{D}=\left(D_{1}+D_{2}\exp\left(D_{3}\sigma^{*}\right)\right)\left(1+D_{4}\ln\left({\dot{\varepsilon }}^{*}\right)\right){\left(1+D_{5}T^{*}\right)}^{-1},$$

where $D_{1},D_{2},D_{3},D_{4},$ and $D_{5}$ are material specific damage parameters, ${\dot{\varepsilon }}^{*}$ is the effective plastic strain rate (3), and $T^{*}$ is the normalized temperature (4). Since the information $D_{c}$ is already implemented in $D_{1}D_{5}$, we set $D_{c}$ in (15) to 1. The JC model proposes an exponential depending on the stress triaxiality, a logarithmic influence of the strain rate, and a linear influence of the normalised temperature.

#### 2.3.2. Lemaitre Fracture Criterion (LEf)

The LEf model goes back to the work of Lemaitre [43] in 1985 and is based on elastic strain energy to failure. It suggests

(19) $$\dot{D}=\frac{1}{D_{c}}{\left(-\frac{Y}{S}\right)}^{t}$$

with

(20) $$-Y=\frac{\sigma_{eq}^{2}}{2E{\left(1-D\right)}^{2}}\left(\frac{2}{3}\left(1+\bar{\nu }\right)+3\left(1-2\bar{\nu }\right){\left(\frac{-p}{\sigma_{eq}}\right)}^{2}\right)$$

where $\sigma_{eq}$ is the equivalent von Mises stress, ν is the Poisson’s ratio, and $D_{c},E,S$ and *t* are material parameters. Here, $D_{c}$ is a material parameter and not necessarily equal to one. It has been demonstrated to be a well-suited model for the application of high-velocity impact [21].

#### 2.3.3. Fracture Criterion Due to Dolinski and Rittel (DRf)

Dolinski and Rittel propose an energy failure criterion based on plastic work [44,45,46,47]. They define a critical level of plastic strain energy density

(21) $$W_{crit}=\int_{0}^{{\bar{\varepsilon }}_{p}^{\mathrm{crit}}}\sigma_{eq}d{\bar{\varepsilon }}_{p},$$

where ${\bar{\varepsilon }}_{p}^{\mathrm{crit}}$ is a material specific level of plastic strain at which the structural strength starts to deteriorate. In this model, gradual element failure

(22) $$\sigma_{eq}^{*}=\sigma_{eq}\left(1D^{b}\right).$$

where *b* describes the amount of softening, is a substantial part, as the reduced yield strength is required in the computation of the plastic strain energy density (21). For the JCf and LEf model, the coupling is described in Section 2.5. The damage evolution is

(23) $$D=\left\{\begin{array}{cc} 0 & W\leq W_{\mathrm{crit}}\\ \frac{W-W_{\mathrm{crit}}}{W_{\mathrm{frac}}-W_{\mathrm{crit}}} & W>W_{\mathrm{crit}} \end{array}\right.,$$

where $W_{\mathrm{frac}}$ is the plastic strain energy density at which the stress drops to zero, and

(24) $$W=\int_{0}^{{\bar{\varepsilon }}_{p}\left(t\right)}\sigma_{eq}^{*}d{\bar{\varepsilon }}_{p}.$$

Considering the definition of the fracture parameters, this fracture criterion is very descriptive. The user only needs to specify ${\bar{\varepsilon }}_{p}^{\mathrm{crit}}$ and $W_{\mathrm{frac}}$.

### 2.4. Damage Accumulation under Different Triaxialities

The fracture characterization test results by Bao and Wierzbicki [48] “proved conclusively that there is a cutoff value η=−1/3 below which the fracture never occurs no matter what the magnitude of the equivalent strain may be”. This criterion can be directly applied to all prior described damage models, and we show in Section 4.5 that modifying the LEf and DRf model with this criterion is essential to obtain qualitatively and quantitatively better accordance with the experimental results for our application.

### 2.5. Modifying the Yield Strength Due to Damage

The motivation for modifying the yield strength not only for the DRf model (compare (22)) is to improve the description of material weakening during the necking phase. The softening factor due to damage $C_{D}$ is defined by

(25) $$\sigma_{y}=C_{D}\sigma_{y}^{0}\;\;\mathrm{with}\;C_{D}=1-{\left(D/D_{c}\right)}^{1/c},$$

where *c* is a material specific softening parameter analogous to Dolinski et al. [44]. The material weakening of initial yield strength $\sigma_{0}$ due to damage is a similar mechanism like temperature softening. Therefore, we implement it in the plasticity model in the same way:

In the JC model, we multiply $C_{D}$ as a fourth factor in (2), resulting in another modified JC model

(26) $$\sigma_{y}\left({\bar{\varepsilon }}_{p},{\dot{\bar{\varepsilon }}}_{p},T\right)=\left(A+B{{\bar{\varepsilon }}_{p}}^{n}\right)\;\left(1+C\ln({\dot{\varepsilon }}^{*})\right)\;\left(1-{\left(T^{*}\right)}^{m}\right)C_{D}.$$

In the RK plasticity model, the temperature reduction affects directly the Young’s modulus (9). In terms of damage softening, we assume that the Young’s modulus is also a function of damage

(27) $$\frac{E(T,D)}{E_{0}}=C_{D}\left(1-\frac{T}{T_{m}}\exp\left(\frac{T_{ch}}{T_{m}}\left(1-\frac{T_{m}}{T}\right)\right)\right).$$

Since the RK plasticity model suggests that the elastic modulus is proportional to the material weakening, we decided to also soften the Young’s modulus for the JC and CS model:

(28) $$E=C_{D}E_{0}.$$

**Figure 2:**
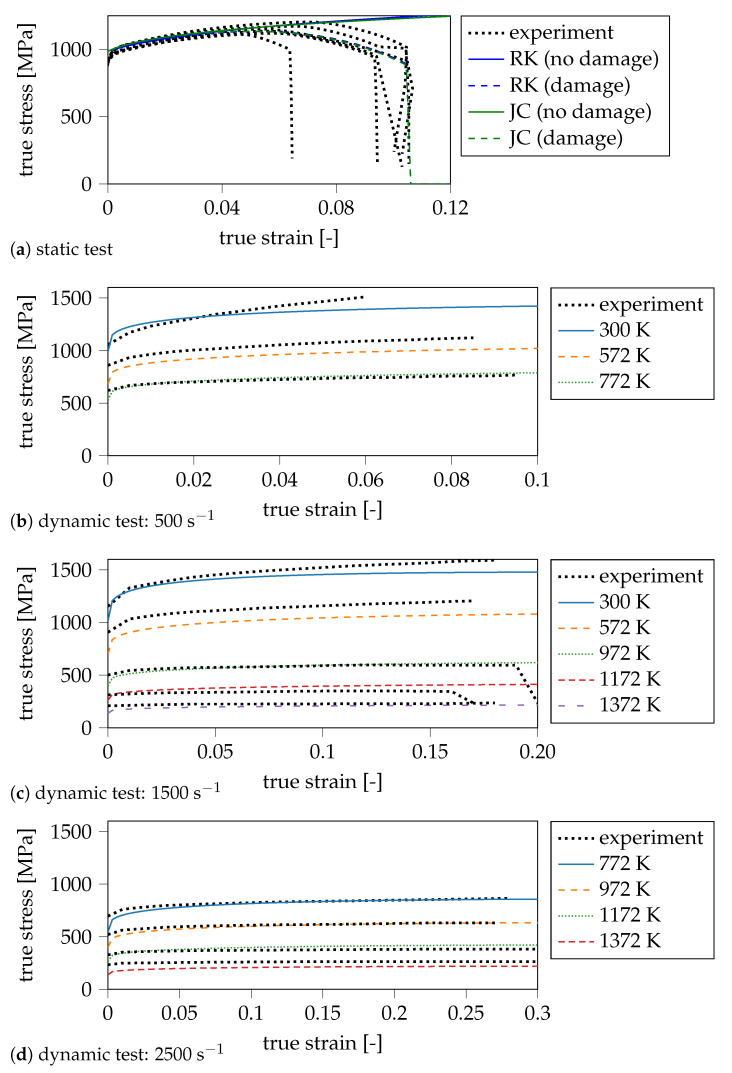
Material calibration: the red dotted lines show the experimental results. (**a**) fit of static constants for the JC and RK model with damage (dashed lines) and without damage (solid lines); (**b**–**d**) fit of dynamic parameters (JC model) for different strain rates (500 s^−1^ to 2500 s^−1^) with data of Lee and Yeh for temperatures between 300 K and 1372 K [39].

This affects the elastic predictor that uses the shear modulus, which is an invariant of *E*. The next section presents the material calibration of the applied 4340 steel. This section further clarifies the necessity of including necking in the plasticity model (compare Figure 2a).

### 2.6. Parameter Estimation for the JC and RK Plasticity Model

In this section, we show the calibration of the plasticity model parameters for the ring steel in our experiments (4340 steel); parameters for the RK model are not available and both JC and RK models have to be calibrated to the same curves for consistency. The input constants for the plasticity model are (i) properties that are general for steel, (ii) numerical parameters, (iii) parameters that describe the state for static loading, and (iv) parameters describing the dynamic loading. Values (i) are found in literature [49], and (ii) are defined based on the numerical problem (e.g., the range of strain rates). Thus, we determine the material constants (iii) and (iv).

These parameters are fitted to stress–strain curves obtained by material testing (compare Figure 2). For both plasticity models, we decouple the estimation of static and dynamic parameters. First, the static input values are identified based on tensile tests that were carried out as part of the original investigation [21]. Then, to fit the dynamic material constants, we utilise experimental data of the same type of steel (4340 steel) from the paper of Lee and Yeh [39]. Considering the experimental data of the static tests, obtained at standard conditions, and the extrapolation of the dynamic data to quasi-static conditions, both give the same yield strength; this is an indication that both kinds of steels are similar. Neglecting the dynamic part of the hardening rules at first, we obtain simplified models to fit the static input constants: The JC rule (2) becomes

(29) $$\sigma_{y}\left({\bar{\varepsilon }}_{p}\right)=\left(A+B{{\bar{\varepsilon }}_{p}}^{n}\right),$$

with flow stress *q*, plastic strain ${\bar{\varepsilon }}_{p}$, and static parameters $\sigma_{y},B,n$. The RK model (compare Section 2.2.3) becomes

(30) $$\sigma_{y}\left({\bar{\varepsilon }}_{p}\right)=B_{0}\;2^{-\nu }{\left(\varepsilon_{0}+{\bar{\varepsilon }}_{p}\right)}^{n_{0}},$$

with static input constants $B_{0}$ and $n_{0}$, and the dynamic parameter ν.

As only the static stress–strain curve has to be fitted, there is no need for automation or a hierarchical systematic approach.

**Figure 3:**
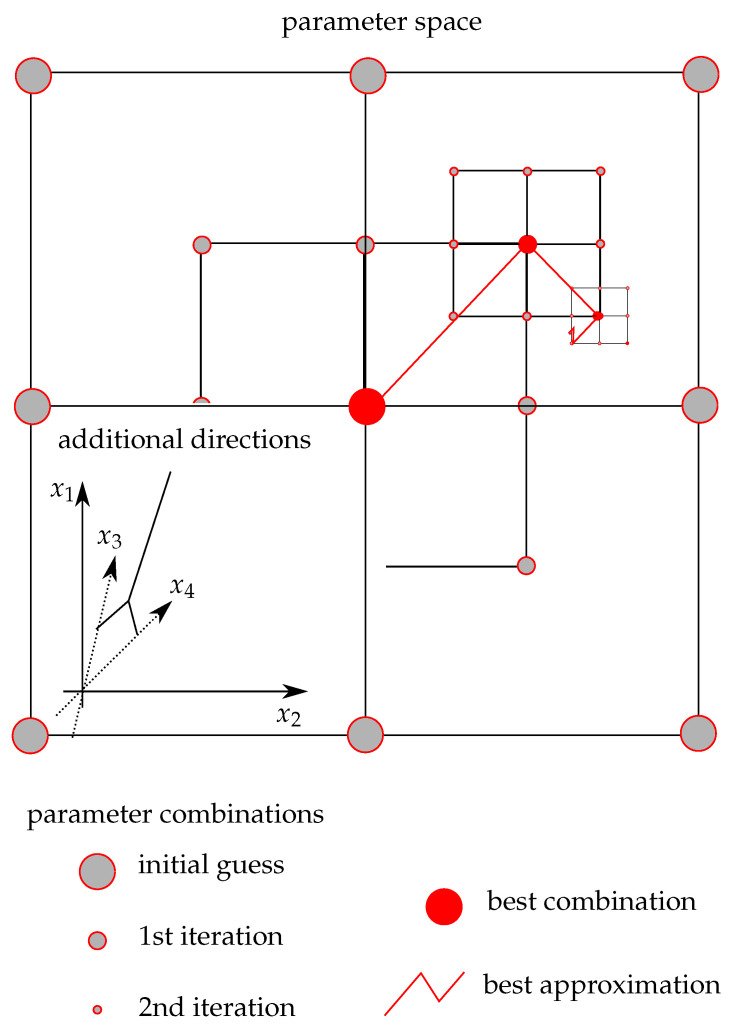
2D schematic of the hierarchical approach used to approximate parameters quickly in high-dimensional parameter space; each dimension represents one parameter $x_{i}$; the algorithm identifies the best fit for the given data (here stress–strain pairs from material tests) (published in [52]).

The dynamic parameters are then determined based on the experimental curves from Split–Hopkinson pressure bar (SHPB) tests [50,51] conducted by Lee and Yeh [39]. The experimental curves and the corresponding fit is shown in Figure 2b–d. For the calibration, we use a least-squares regression on a hierarchical grid. Since the static input constants are already determined, the approximation space is small enough to distribute possible values for the constants on a regular grid (compare Figure 3).

We discretise the 12 experimental curves with 10 data points resulting in 120 fitting points in total. Assuming that the parameter combination with minimal mean-square error on a coarse grid is close to the global minimum, we refine the grid around the minimum of the parent grid and improve the parameter estimate until convergence. For the plasticity model, this approach finds material constants that approximate all twelve curves well. However, it is difficult to say whether the constants determined in the range of 500–2500 s${}^{-1}$ are also accurate for strain rates between $1.0\times 10^{4}$–$1.0\times 10^{6}$ s${}^{-1}$ as observed in our experiments.

The results for the calibration for both plasticity models are presented in Table 1 and Table 2.

### 2.7. Other Modelling Aspects

The numerical model is set up as follows: the discretisation length of the explosive is identical to the cylinder ring to guarantee correct physical interactions resulting in approximately 20,000 particles for the ring and 150,000 for the explosive for the 1:1 case. The spatial resolution is kept constant for the different ring sizes. The material parameters were extracted from quasi-static and dynamic stress–strain curves (see Section 2.6) and are presented in Table 1 and Table 2. In addition to the plasticity model, a Mie–Grüneisen equation of state describes the change in the thermodynamic state with the following set of parameters: speed of sound C=4570 m/s, linear shock parameter $S_{1}=1.4$, and Grüneisen parameter $\gamma_{0}=1.67$. For the explosive, a Jones–Wilkins–Lee equation of state [53,54] describes the expansion during the explosion. The equation of state is combined with a high explosive (HE) material model that describes the Chapman–Jougot pressure $\left(p_{CJ}=292\;\mathrm{Mbar}\right)$, the detonation velocity $(v_{d}=8250$ m/s), the artificial viscosity coefficients $\left(Q_{1}=1.5,Q_{2}=2.0\right)$, and the initial density of the explosive ($\rho_{0}=1740\;\mathrm{kg}/m^{3}$). For the interaction between explosive and steel, no explicit contact algorithm is needed in SPH. The contact is described with the standard kernel interpolation of SPH.

## 3. Case Setup and Review of Experiment

**Figure 4:**
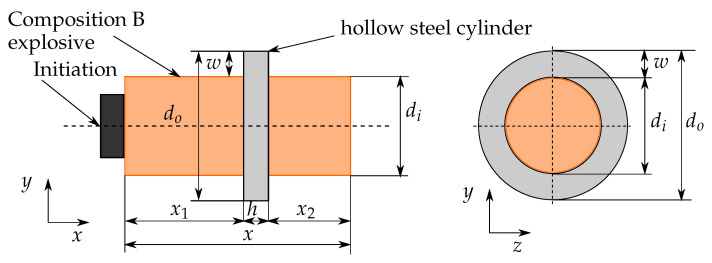
Charge dimensions of the experiment. Constant for all cases: The inner diameter ($d_{i}=38.1$ mm), the outer diameter ($d_{o}=57.2$ mm), the charge extension left ($x_{1}=45$ mm), the charge extension right ($x_{2}=32$ mm), and the wall thickness of the cylinder (w=9.5 mm). (Image similar to [21]).

The experiments are explained in detail by De Vuyst et al. [21]. A hollow steel cylinder (grade 4340) is placed over an explosive charge (Comp B, RDX/TNT 65/35), and the charge is detonated. The detonation pressure leads to a radial acceleration where the ring is subjected to complex loading history, which results in fracture. The study includes four different height to width (h/w) aspect ratios (1:1, 2:1, 3:1, 10:1). Figure 4 illustrates the size of the cylinder ring.

**Figure 5:**
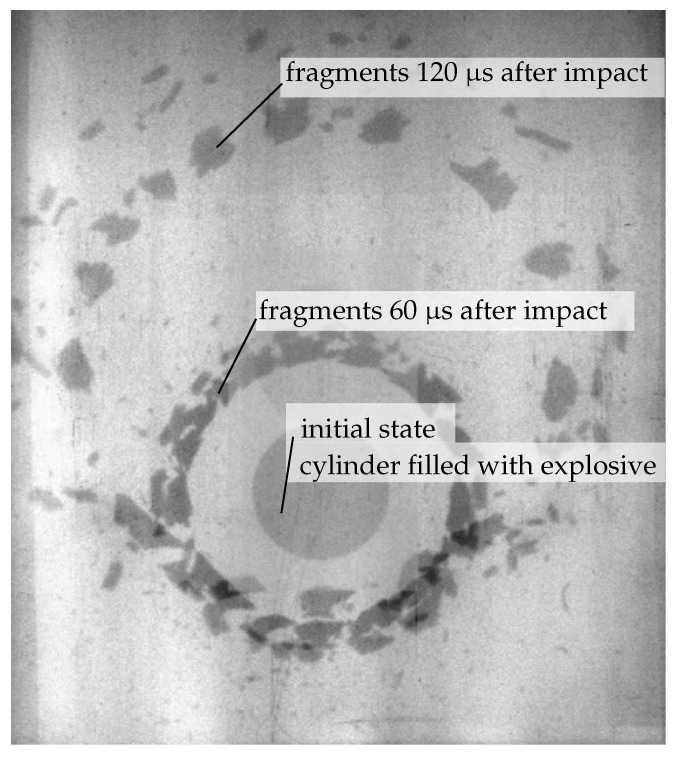
Double-exposed X-ray visualization of ring expansion for 1:2 aspect ratio (exposures 60 μs and 120 μs after detonation).

In the experiment, fragments were recovered in a water basin covering approximately 25% of the cylinder ring. Due to the complexity of the setup, only 60% to 80% of the expected mass was recovered. Particularly for the 1:10 case, the water basin was considerably deformed, and the experimental results have to be interpreted carefully. In addition to the fragment recovery, X-ray reveals the qualitative behaviour (see Figure 5).

## 4. Numerical Results

### 4.1. Overview of the Numerical Simulations

The numerical simulations investigate the influence of the following modelling aspects:

1. the description of material imperfections with a randomised initial damage distribution,
2. the modelling of damage mechanics,
3. the improvement of the accuracy of the plasticity algorithm with an iterative stress solver,
4. the effect of a triaxiality based damage cutoff criterion, and
5. the range of applications for different combinations of plasticity models and fracture criteria and all aspect ratios of the ring.

Each aspect is addressed individually in the following paragraphs. Studies 1–4 investigate the 1:2 rings with the CS plasticity model and the LEf damage model if not further specified.

### 4.2. Randomisation of the Initial Damage

**Figure 6:**
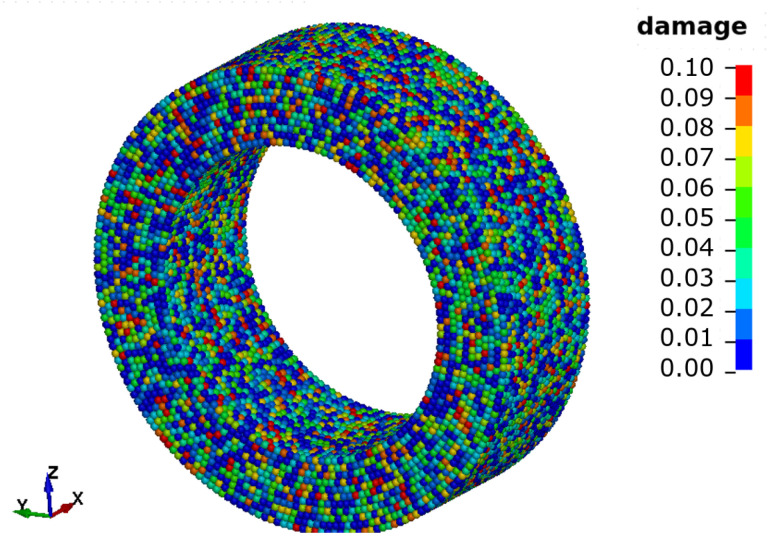
Initial damage distribution for an aspect ratio of 2:1 and $D_{0,\max}=0.1$.

**Figure 7:**
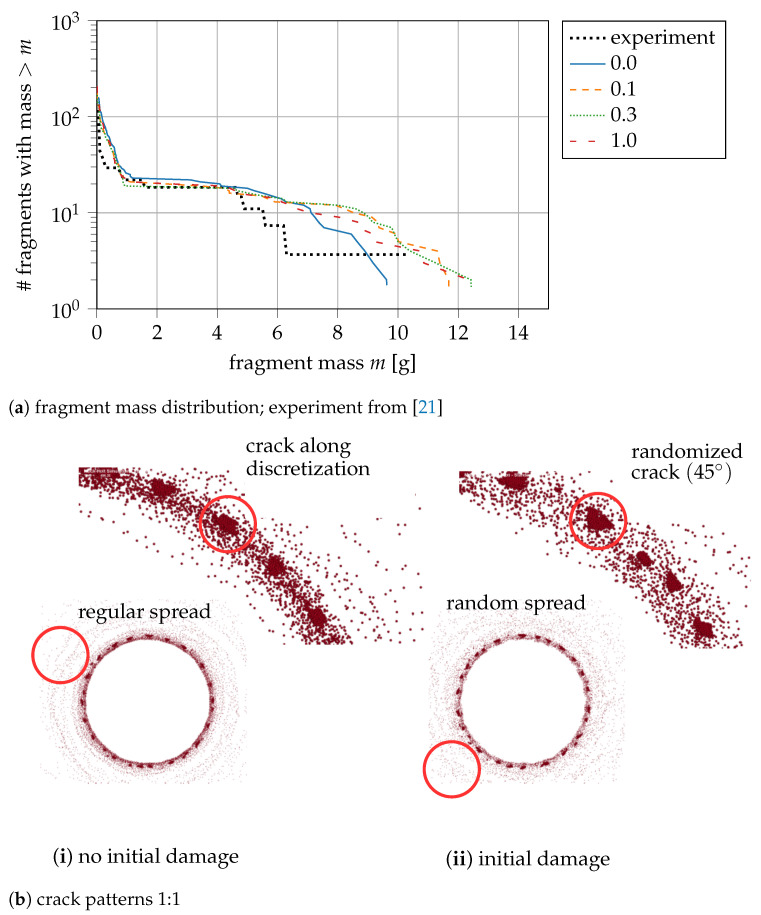
Influence of implementing an initial damage distribution to the fracture behaviour of the hollow cylinder ring; the label “0.0”–“1.0” is the amount of maximum damage *D*_0,max_ during the initial distribution.

We implement a randomised distribution of initial damage to model the imperfections in the material (see Figure 6). These imperfections are assumed to be small and only present for a small amount of material or particles. We calculate the initial damage of particle *i* as

(31) $$D_{0}\left(i\right)=D_{0,\max}\;{\mathrm{rand}(i)}^{2};\;\mathrm{rand}\in \left[0,1\right)$$

where $D_{0,\max}\geq 0$ is the maximum value of initial damage, and rand(i) is an equally distributed random value between 0 and 1. For a non-uniform distribution that favours small values of $D_{0}$, we use the square function in (31). We use the same random seed for each ring size, to compare two simulations with a random initialization. As a default value, we use $D_{0,\max}=0.1$ and compare it with larger initial damage (0.3, 1.0) and no initial damage (0.0) in Figure 7a.

The plot in Figure 7a shows the mass distribution of the fragments at the final state of the simulation. The experimental results are visualised in red and the numerical simulations in blue, orange, green, and dark red. Experiments and numerical simulations are in good agreement in terms of the amount of small and medium-sized fragments (m≤5 g). For large fragments (m>5 g), the simulation predicts a larger number of fragments. With randomised initialisation, the number of large fragments is higher and also the mass of the large fragments is bigger than without initial damage. The qualitative behaviour of the cracks is only described with initial damage correctly (compare Figure 7b). Without randomization of initial damage, artificial crack patterns occur. On the contrary, the randomization of initial damage leads to crack patterns as they are found for the fragments of the experiments. This is an important attribute of our numerical model, and we identify a suitable value of $D_{0,\max}$ in the following. Considering the curves with random damage initialisation in Figure 7a, we observe that an increase of the initial damage parameter from 0.1 to 0.3 has negligible influence, while a more significant difference can be observed for $D_{0,\max}=1.0$. A value of 0.1 for initial damage is sufficiently small to not lead to earlier crack formation compared to the model without random damage initialisation, yet it is large enough to give similar fracture patterns to a larger initial damage value. The randomisation avoids preferred crack propagations and models the imperfections as required. Small initial damage does not provoke an earlier fracture of the ring.

### 4.3. Coupling of Plasticity and Fracture Model

**Figure 8:**
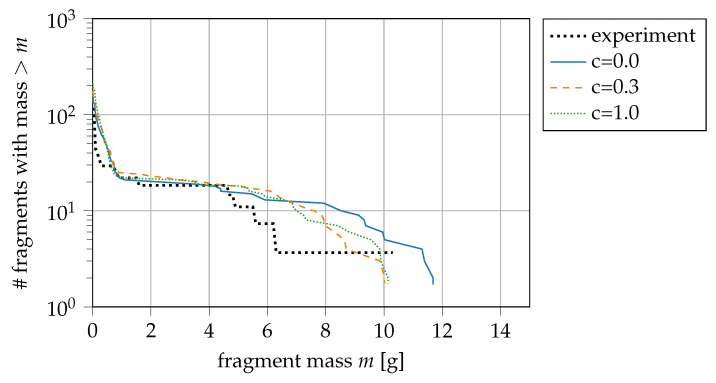
Coupling of plasticity and fracture model: *c* = 0 is no coupling, *c* = 0.3 is best fit, and *c* = 1.0 is the description in classical damage mechanics; experiment from [21].

As observed in the static stress–strain curves, a coupling of plasticity and fracture model to describe the reduced yield strength due to damage improving the hardening curve shape (compare Figure 2a). We recall the coupling function (25), where $C_{D}$ is defining the reduction in yield strength $\sigma_{y}$ due to damage *D* with the constant *c* and the initial yield strength $\sigma_{y}^{0}$. The parameter *c* according to static stress–strain curves of our material is c=0.3. This is in agreement with the analysis of Dolinski et al. [55]. Figure 8 shows the influence of the coupling in the ring experiments. Three parameters are chosen for *c*: a simple failure criterion (c=0.0), the experimental fit described before (c=0.3), and (c=1.0) as it is implemented in classical damage mechanics. Dolinski and Rittel [44,47] also suggest c=1 for most applications. In terms of our application, the necking only affects the mass of the largest fragment. The material behaves softer for strongly damaged particles when the coupling is activated. Otherwise, these particles might stay attached, leading to larger fragments. While the largest fragment is 20% heavier compared to the experiment, the simulation with coupling activated predicts the equivalent mass. The coupling with the fitted value (c=0.3) predicts less large fragments (m > 5 g), c=1 does not change the fragment statistics compared to c=0.3. For our application, we conclude that enabling the coupling, as suggested by the experimental data, has a positive but small impact on general behaviour and is obligatory to describe the material correctly.

### 4.4. Newton–Raphson Iterative Solver in the Plasticity Algorithm

**Figure 9:**
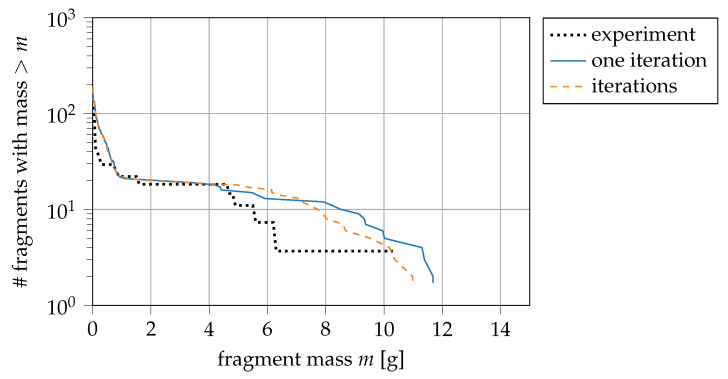
Influence of using several Newton–Raphson iterations instead of only one iteration; experiment from [21].

The plasticity algorithm predicts the stress update by back projecting the elastic predictor’s stress state to the yield surface. This constitutes a nonlinear system of equations. To minimise the projection’s error, we can use an iterative predictor-corrector scheme developed by Key and Krieg [56,57] and further developed by Simo et al. [58]. The iterative approach increases the runtime compared to a “one-step prediction” commonly implemented in commercial code for performance reasons (e.g., Nemat-Nasser [59]). However, particularly for large strain increments, an iterative approach also improves the stress approximation precision. The one-step prediction that we apply returns the deformation based on one iteration of the iterative approach. The iterative approach calculates several iterations which use a cutoff criterion to determine convergence that evaluates the relative difference between equivalent stress and flow stress. Our study defines the relative cutoff of $1\times 10^{-5}$, which is generally reached with less than five iterations. Due to the highly dynamic expansion of the cylinder rings, we assume large strain increments. On the other hand, the time step that is calculated by the minimum distance of particles and the speed of sound in the material—in the explicit time integration—partly compensate for this. The question to be answered is whether an iterative approach influences the solution or not. Figure 9 shows that the iterative approach does not significantly impact the result, but it shows a tendency in the right direction (better match with experimental data).

### 4.5. Modifications of the Fracture Criterion

In the previous comparisons, we have seen that (i) damage initialisation, (ii) coupling, and (iii) a modification of the plasticity algorithm have only little influence on the result. In this section, we show that the damage model is the primary driver of the fractures. This result is essential as we can focus on the damage model and do not expect significant changes when using modifications proposed above.

Our LEf model is modified with two additional criteria [43,60]. First, a widely accepted triaxiality cutoff criterion by Bao and Wierzbicki [48] prevents damage accumulation for (η<−1/3). Second, damage is only accumulated above a defined threshold strain ${\bar{\varepsilon }}_{p}<{\bar{\varepsilon }}_{p}^{t}$. The original model is retained by setting ${\bar{\varepsilon }}_{p}^{t}=0.0$.

**Figure 10:**
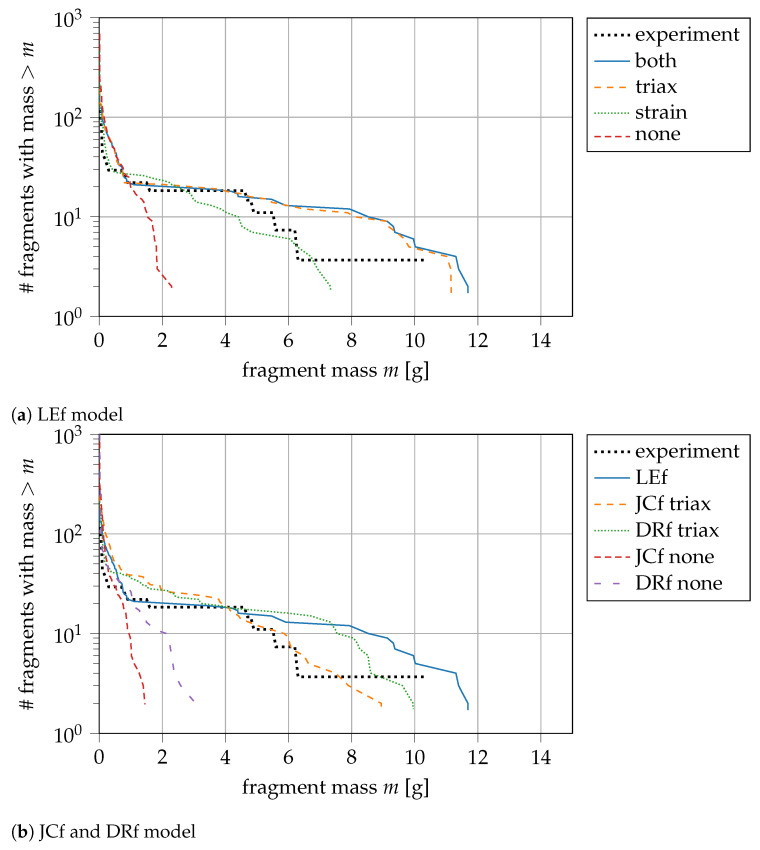
Modifications of the damage criterion for LEf, JCf, and DRf, results with the triaxiality cutoff criterion are much closer to the experiments for all criterias; experiment from [21].

In Figure 10a, we investigate four cases: the baseline result with both criteria (both), one case with only a triaxiality criterion (triax), one using only a plastic strain criterion (strain), and one without any modification (none). The latter case is not able to reproduce the experiments at all. The fragments are much smaller as they scatter already in a very early stage of the simulation. Using only the plastic strain criterion coincides with the experiment except for the largest fragment. The baseline result (both) and (triax) predict the same statistical distribution. Both overpredict the number of large fragments but are closer to the experiment regarding the largest fragment mass. Since the two results are identical, we conclude that the triaxiality criterion is stricter than the plastic strain criterion. This result might be directly related to our application: the pressure of the expanding explosive dominates the stress state in the beginning. When the pressure drops and the ring is radially accelerated, tensile and shear stresses occur. At this stage, the plastic threshold strain of 0.1 is already reached.

In a second step, we add the triaxiality criterion also to our implementation of the JCf and DRf model. We observe the same scattering as for the LEf model (red and purple curve in Figure 10b) if no modification of the damage model is implemented. Applying the triaxiality criterion results in a distribution which is much closer to the experimental findings and enhances the applicability of the model (compare Figure 10a,b). Therefore, it is applied to all fracture models in the final study presented in the following paragraph.

### 4.6. Plasticity and Fracture Models

**Figure 11:**
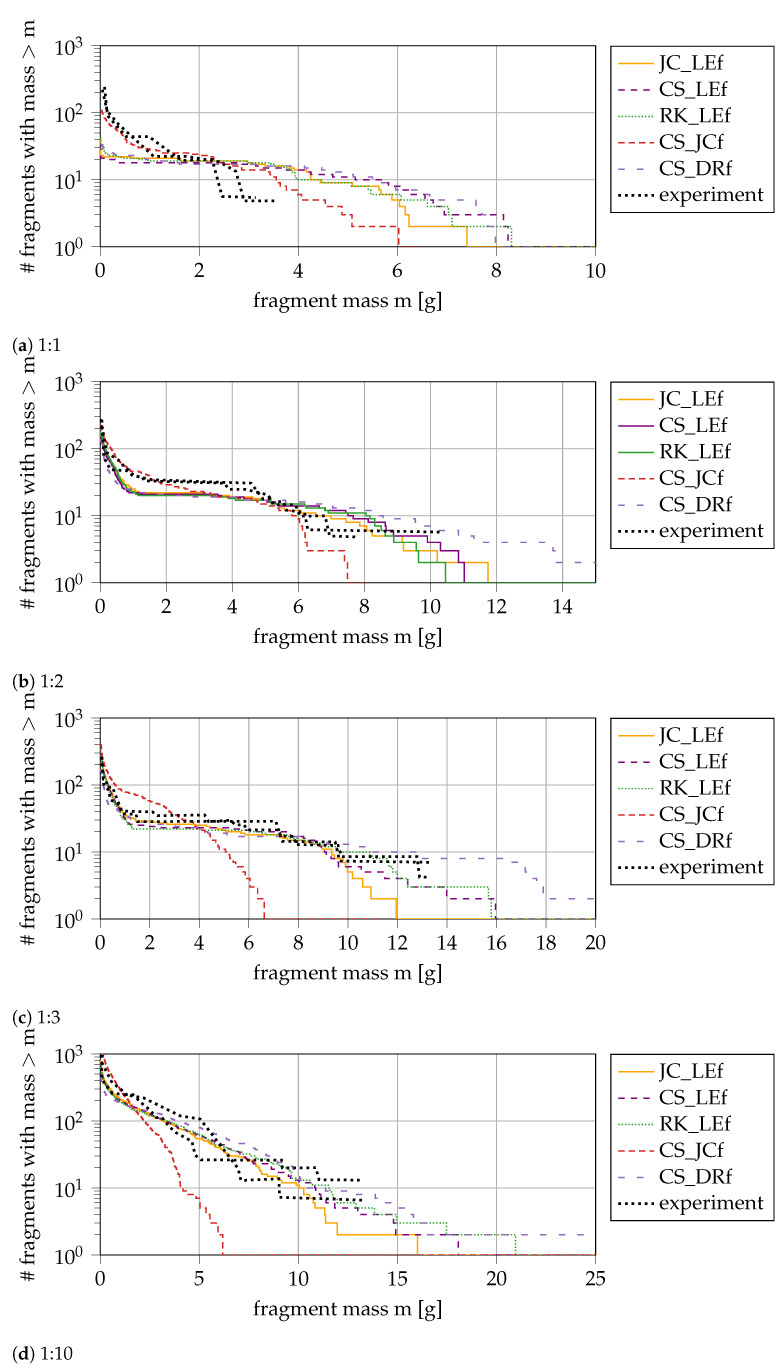
Fragment mass distribution for different combinations of hardening rule and damage model: JC_LEf = JC hardening + LEf damage, CS_JCf = CS hardening + JCf damage, CS_LEf = CS hardening + LEf damage, CS_DRf = CS hardening + DRf damage, RK_LEf = RK hardening + LEf damage; experiment from [21].

We have quantified the influence of all modifications implemented in our numerical model and determined suitable settings to describe material imperfections and damage. In this final study, we apply our model to different cylinder sizes and compare three hardening rules (JC (blue), CS (yellow), and RK (green)), and three fracture models (JCf (black), LEf (yellow), and DRf (purple)) against each other (see Figure 11). Based on previous findings, we expect that the differences due to the fracture models predominate. Each subplot of Figure 11 shows a different ring size (1:1, 1:2, 1:3 and 1:10). In the following, we use the default values determined above (initial damage distribution 0.1, reduction of the yield strength with (*c* = 0.3), and triaxiality cutoff activated). The experimental results (red) are the references for our validation. Based on the statistical errors of the few experiments, the focus is a qualitative comparison rather than a quantitative. Up to twice as heavy fragments are predicted by the numerical model than the experiment. The largest deviation from experimental data is found for the smallest ring size.

We can see small differences between the numerical data regarding the plasticity model, best visible for an aspect ratio of 1:3. Regarding the fracture model, we observe more significant differences than between the plasticity models: For 1:1 and 1:2, the numerical models’ mass distribution is similar, while, for 1:3 and 1:10, the fracture models predict different results with the same parameters. As the change of plasticity model does not influence the mass distribution, we conclude that some fracture models do not correctly capture all fracture mechanisms or need further parameter calibration. Only the JCf model is not predicting the experiments for large aspect ratios of the ring correctly. It underpredicts the fragment mass for the 1:3 and 1:10 case. In contrast to the inaccurate prediction of the JCf model for the large fragments, it is more accurate in the number of small fragments (m<2 g) for the 1:1 and 1:2 case. The JCf model parameters are more difficult to determine than the DRf and LEf parameters because the JCf model contains more input parameters than the other two, which are less descriptive and need more calibration data. We conclude that the parameters we identified for the JCf model can only describe a small range of our application cases. The DRf and LEf model, on the other hand, are consistent in the fragment mass prediction: for both, cracks occur a few microseconds later than in the experiment, resulting in larger fragments.

## 5. Conclusions

We investigated five modifications of a numerical model to describe the natural fragmentation of cylinder rings better, which can be transferred to the application of ballistic impact: (i) a randomised damage initialisation to describe material imperfections, (ii) a use of a damage model, instead of a fracture criterion, to capture the strain softening part of the stress–strain curve, (iii) an iterative plasticity radial return algorithm to improve accuracy, (iv) adding a triaxiality cutoff criterion to the damage models, and (v) different constitutive models (hardening rules) to determine the most suitable model. Our results demonstrate that, in particular, the inclusion of a triaxiality cutoff criterion in the damage models, which allows damage accumulation only for η>−1/3, is essential for highly dynamic scenarios, such as ballistic impact. The LEf (fracture model due to Lemaitre) and the DRf (fracture model due to Dolinski and Rittel) model modified with the triaxiality cut-off criterion described the ring’s breakup for all ring sizes correctly, while the JCd (fracture model due to Johnson and Cook) did not reproduce the fracture behaviour for larger aspect ratios. The damage initialisation is also important, as it improves the triggering of random crack patterns, and results in more realistic fragment shapes. Finally, the strain softening introduced by the coupling of damage and plasticity models and the iterative plasticity algorithm also has a positive effect on the fragment mass distribution, but this effect is secondary. In terms of the choice of the hardening rule, we observed small differences.

The determination of the material parameters was done as part of this work. While we found literature data to calibrate the plasticity models, we used the ring experiments’ qualitative results to determine damage parameters. This was easiest for the DRf model since it contains only three illustrative parameters. For the LEf model, we set the parameters determined in the original study. For the JCf model, it was not possible to determine suitable values for the five parameters applicable for all tests.

Our approach, using a simplified experiment, allowed us to isolate the study of damage parameters from other influences such as the contact modeling required for ballistics. The damage parameters and the modifications of the model determined in this study are applied for ballistic applications in the future to verify the benefits of the suggested model choices.

## Figures and Tables

**Table 1:** Estimated JC and CS parameters: static behaviour determined according to test data [21] and dynamic behaviour, according to Lee and Yeh [39]. The values are converted from the test strain rate to the reference strain rate of 1 s${}^{-1}$.

Parameter	JC Paper	JC Fit	CS Fit	Comment
*A* [MPa]	792	880	880	static parameter
*B* [MPa]	510	833	830	static parameter
*n* [-]	0.26	0.26	0.26	static parameter
*m* [-]	1.05	0.75	0.75	thermal softening
*C* [-]	0.014	0.025	-	strain rate parameter
$\bar{C}$ [s${}^{-1}$]	-	-	$9\times 10^{6}$	strain rate parameter
$\bar{p}$ [-]	-	-	5	strain rate exponent

**Table 2:** Estimated RK parameters determined: static behaviour according to test data [21] and the dynamic behaviour, according to Lee and Yeh [39].

Parameter	Value	Determined By
$B_{0}$ [MPa]	1600	static experiment (scaling)
$n_{0}$ [-]	0.12	static experiment (curvature)
$\varepsilon_{0}$ [-]	0.018	numerical parameter
${\tilde{D}}_{1}$ [-]	0.49	general for steel
ν [-]	0.225	dynamic experiment
$\sigma_{0}^{*}$ [MPa]	352	dynamic experiment
*m* [-]	1.10	dynamic experiment
${\tilde{D}}_{2}$ [-]	0.0108	dynamic experiment
$E_{0}$ [GPa]	212	general for steel
$\theta^{*}$ [-]	0.59	general ferritic steel
$T_{m}$ [K]	1600	general for steel
${\dot{\varepsilon }}_{\max}$ [$s^{-1}$]	$10^{7}$	numerical parameter (defined)
${\dot{\varepsilon }}_{\min}$ [$s^{-1}$]	$10^{-5}$	numerical parameter (defined)
$C_{p}$ [J ${\mathrm{kg}}^{-1}$ $K^{-1}$]	470	general for steel
β [-]	0.9	general for steel
$\rho \;[\mathrm{kg}\;m^{-3}]$	7800	general for steel
$\alpha \;[K^{-1}]$	$10^{-5}$	general for steel

## Data Availability

Not applicable.
